# Connection Mechanism of Molten Pool during Laser Transmission Welding of T-Joint with Minor Gap Presence

**DOI:** 10.3390/ma11101823

**Published:** 2018-09-25

**Authors:** Ke Xu, Haichao Cui, Fuquan Li

**Affiliations:** 1 Shanghai key Laboratory of Laser Processing and Materials Modification, School of Materials Science and Engineering, Shanghai Jiao Tong University, Shanghai 200240, China; xuke@baosteel.com; 2 State Key Lab of Advanced Welding and Joining, Harbin Institute of Technology, Harbin 150001, China; lifuquan@hit.edu.cn

**Keywords:** T-joint laser welding, minor gap, simulation, pores

## Abstract

Laser welding of T-joint transmitting from the face plate to the core is commonly used in the sandwich structure preparation. Minor gaps between the face and the core plate are inevitably present after several beads on the sandwich structure welding due to the thermal deformation. The effects of gap presence on fluid flow from the face to the core plate are rather significant, where the gas can be easily entrapped into the pool and form the pores. To this end, three-dimensional transient simulations based on VOF (volume of fluid) method were conducted to explore and ascertain the effect of fluid flow inside the pool on the pore formation due to the gap presence. It was found that minor gap within 0.2 mm will not reduce the welding quality. Under the effects of gravity and surface tension, the fluid from the face sheet will drop down to the core, which removes all the air out of the gap and the laser goes through the fluid of the gap and then shines on the core, which prevents the air from being entrapped into the pool. While the laser goes though gap, the wall of keyhole opens and closes continuously. The vibrating time of keyhole is approximately 0.029 s. After finishing the vibration, the welding is stable, which is the same as common unfull penetration. Finally, the simulated results are well verified through observing the plasma oscillating frequency in the gap and comparing to the pore-free bead profile. This paper supplies evidence that minor gap presence during laser transmitting welding on sandwich structure has nothing to do with pore formation.

## 1. Introduction

With the development and prevalence of joining technologies such as laser and electron beam, the joining of some new structure such as sandwich structure can be realized and applied in the shipping, train and truck industry. Sandwich structure is widely used not only for saving material to replace thick plate but also for its high strength to weight ratio. Jukka et al. [1] reported that different sandwich structures were designed such as I-core with straight webs, O-core with rectangular beams and other structures according to demands of stiffness and strength. When the sandwich structure is assembled and welded, a gap between face-sheet and core plate is inevitable due to the thermal deformation after multi pass welding. Experimental research has shown that the gap presence, where the air is easily involved and pores are formed in the weld bead, would produce an important effect on the weld performance, especially on pore formation [2,3,4]. Besides, the laser energy loss with the form of plasma in the gap is large and would affect the penetration depth. In previous reports, most researchers emphasized on evaluating the mechanical properties such as fatigue, bending, stiffness, etc. [5,6,7,8,9]. Few researchers paid attention to the molten pool performance under the condition of gap presence, although the pool thermal field has been simulated [10]. Therefore, the connecting mechanism of pool between the face and the core has to be considered. The mechanism of molten pool formation between face and core plate is extremely complicated under the condition of gap presence, such as how to fill out the gap and connect between face and core plate, especially whether the laser or fluid first touching the core plate through the gap leads to air in the pool. If these questions are clearly answered, welding quality can be easily controlled for the joint with the gap presence. In fact, experimental observation and measurement have difficulty solving this problem because the keyhole is surrounded by fluid and the laser cannot be seen through the fluid. Some researchers have calculated and simulated the welding deformation on the sandwich structure [11]. However, on the molten pool behavior, especially how to the fluid filling into the gap, research is scant. In our previous research [12], the effects of gap size on the pore formation and welding quality is deeply studied by observing the longitudinal weld section, but the connecting mechanism is not referred. To investigate the forming mechanism of pores during laser welding of T-type joint with gap presence, the connecting mechanism of molten pool between face-sheet and core was studied with the help of simulated method. In this research, the gap size of 0.2 mm was chosen to study the forming mechanism of molten pool. The physical model of laser welding I-core sandwich structure was built and analyzed. The connecting mechanism and the effects of gap presence on the pore formation were simulated and discussed. Finally, the simulated results were verified through comparing with the plasma vibrating frequency and cross-sectional profile of welding bead.

## 2. T-Joint Laser Welding Model

**Figure 1:**
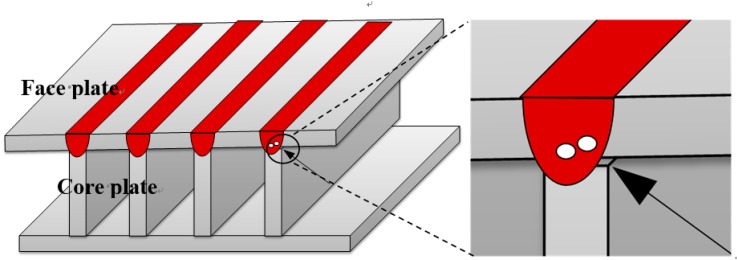
Schematic of sandwich structure.

Figure 1 shows the three-dimensional profile of inevitable present gap after multi pass welding in the sandwich structure due to the thermal deformation. The three-dimensional model was built under this condition and only one T-type joint with 0.2 mm gap size was simulated to save computing time. The differential equations governing the conservation of mass, momentum, and energy based on continuum formulation are as follows.

Momentum equation:

(1) $$\rho (\frac{\partial V}{\partial t}+V\cdot \nabla V)=\mu \nabla^{2}V-\nabla P+R_{SOR}\cdot V+F-KV\;$$

Continuity equation:

(2) $$\frac{\partial \rho }{\partial t}+\nabla \cdot (\rho V)=R_{SOR}\;$$

Energy equation:

(3) $$\rho (\frac{\partial E}{\partial t}+V\cdot \nabla E)=\nabla \cdot (k\nabla T)+E_{SOR}\;$$

where

(4) $$E=\int_{T}c(T)dT+(1-f_{s})h_{sl}\;$$

where $R_{SOR}$ is a mass source, V is molten metal velocity, P is hydrodynamic pressure, μ is dynamic viscosity, F is body force (e.g., gravity, recoil force and buoyancy forces), ρ is the fluid density, E is internal energy per unit mass, k is thermal conductivity, T is a local temperature, $E_{SOR}$ is an energy source term due to mass source term, k is the drag coefficient for porous media model, $f_{s}$ is solid fraction and c(T) is specific heat.

VOF method was used to track the free fluid surface. According to kinematic motion equation, the VOF function moves according to the velocity field in the fluid, as shown by Equation (5).

(5) $$\frac{\partial F}{\partial t}+\nabla \cdot (VF)={\overset{•}{F}}_{s}\;$$

For a single incompressible fluid, $\overset{}{F}$ represents the volume fraction occupied by the fluid. If the fluid totally occupies the mesh, $\overset{}{F}$ = 1, and void regions correspond to locations where $\overset{}{F}$ = 0. ${\overset{•}{F}}_{s}$ represents the change of volume fraction of fluid associated with the mass source $R_{SOR}$.

The surface tension γ with the relation of temperature is calculated as follows:

(6) $$\gamma =\gamma_{0}+k_{sur}(T-T_{0})\;$$

where $\gamma_{0}$ is the surface tension at $T_{0}$ and $k_{sur}$ is the temperature coefficient of surface tension.

The recoil pressure is proportional to the saturated vapor pressure $P_{s}(T_{s})$, which in turn depends on the melt surface temperature $T_{s}$ (including the keyhole wall) and internal energy per unit mass U, and can be expressed as follows:

(7) $$P_{r}=AP_{s}(T_{s})=AB_{0}T_{s}^{-1/2}\exp(-\frac{U}{T_{s}})\;$$

Buoyancy is expressed as follows according to the Boussinesq approximation:

(8) $$F_{b}=-\rho g\beta (T-T_{1})\;$$

where ρ is the base metal density and β is the thermal expansion coefficient of base metal.

The temperature boundary at the welding top surface is described in formulation:

(9) $$-k\frac{\partial T}{\partial n}=-q_{1}+h_{c}(T-T_{0})+\sigma \varepsilon (T^{4}-T_{0}^{4})\;$$

For other surfaces:

(10) $$-k\frac{\partial T}{\partial n}=h_{c}(T-T_{0})\;$$

where k is the thermal conduction, $h_{c}$ is the convection coefficient, σ is Stefan–Boltzmann constant, and ε is emissivity.

At first, the laser heat source interacts on the outside plane and then gradually enters the material under the keyhole interaction. Hence, Gaussian plane heat model is used as laser power distribution.

(11) $$q_{1}=\frac{3\alpha Q}{\pi r_{1}{}^{2}}\exp(-\frac{3r^{2}}{r_{1}^{2}})\;$$

where α is laser absorption coefficient, $r_{1}$ is laser radius and Q is laser power.

To model the solid–liquid phase change, the following model about the enthalpy–temperature relation is given:

(12) $$h=\left\{ \begin{array}{l} \rho_{s}C_{s}T\;\;\;\;\;\;\;\;\;\;\;\;\;\;\;\;\;\;\;\;\;\;\;\;\;\;\;\;\;(T\leq T_{s})\\ h(T_{s})+h_{sl}\frac{T-T_{s}}{T_{l}-T_{s}}\;\;\;\;\;\;\;\;\;\;\;\;\;\;\;\;\;(T_{S}<T\leq T_{l})\\ h(T_{l})+\rho_{l}C_{l}(T-T_{l})\;\;\;\;\;\;\;\;\;\;\;\;(T_{l}<T) \end{array}\right.\;$$

where h is enthalpy, $\rho_{s}$ and $\rho_{l}$ are solid and liquid density, respectively, $C_{s}$ and are specific heat at the constant volume of the solid and liquid phases, $T_{s}$ and $T_{l}$ are solidus and liquidus temperatures of the metal alloy, and is the latent heat of fusion for phase change between liquid and solid.

The material used in the simulation is Fe-0.5 mass% C steel. The thermal physical parameters are shown in Table 1. In the simulation, the laser output power is 8 kW and the face-sheet and core plate are both 4 mm thick. The parameter of laser absorption coefficient is 0.85 and the other one used in the simulation is the same as in Reference [12]. The welding speed is 1 m/min. CO_2_ laser welding system (Trumpf, Ditzingen, Germany) was employed to simulate and experimental verification. The laser wave length is 10.4 µm and the focused diameter of laser spot is 0.75 mm. Maximized output laser power is 10 kW. After the connecting between face-sheet and core is finished, the calculation is also ended. Two experiments were conducted to verify the simulation. One used a high-speed camera (Vision Research Inc., Wayne, NJ, USA) (up to 3000 Hz) to capture the plasma in the gap for verifying the keyhole open frequency. The other use weld cross-section comparison to verify the pores present.

## 3. Results and Discussion

### 3.1. Effect of Gap Presence during Welding T Type Joint on the Molten Pool Formation

**Figure 2:**
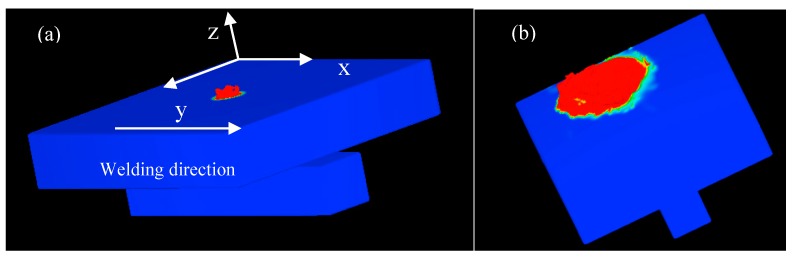
The three dimensional simulated profile with the gap of 0.2 mm: (**a**) t = 0.01 s; and (**b**) t = 0.02 s.

Figure 2 shows the macro profiles of molten pool in simulating T-joint structure plate welding of three dimensions. The area of surface fluid gradually increases as time increases.

**Figure 3:**
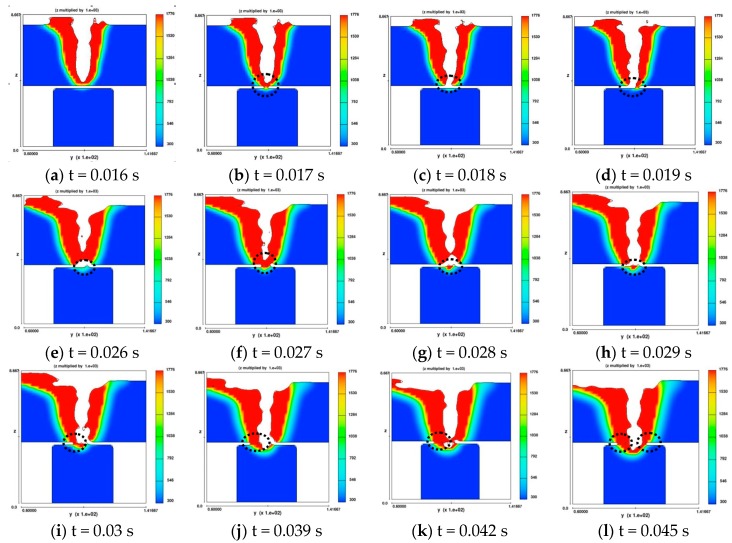
The pool connecting mechanism in the gap location along the x-z plane.

Figure 3 shows two-dimensional profile of molten pool along the cross-section in simulating T-joint structure plate welding when the gap size is 0.2 mm. The cross-sectional profile of molten pool in y-z plane is supplied to explore the connecting mechanism and whether the fluid or the laser first, through the gap, touches the core plate. The full penetration welding was realized on the face sheet at t = 0.016 s, and then the core plate was preheated due to the thermal radiation. At t = 0.017 s, a little fluid touches the core plate but it disconnects immediately at t = 0.018 s due to the gravity and surface tension. Then, the keyhole is fully open and laser directly touches the core plate. While the fluid around the keyhole side wall gradually drops down and connects the core plate, the gap is gradually filled in. Due to gravity and surface tension, the dropped fluid disconnects from the pool until the dropped fluid piles up to the gap size of 0.2 mm. Thus, in this process, the keyhole was fully open and belonged to full penetration welding. The air is not entrapped into the pool due to the fully open keyhole [13]. At t = 0.045 s, the gap is not present, unfull penetration welding was stable and the effects of gap on the pore formation could be neglected. After that, the welding mode is the same as unfull penetration.

**Figure 4:**
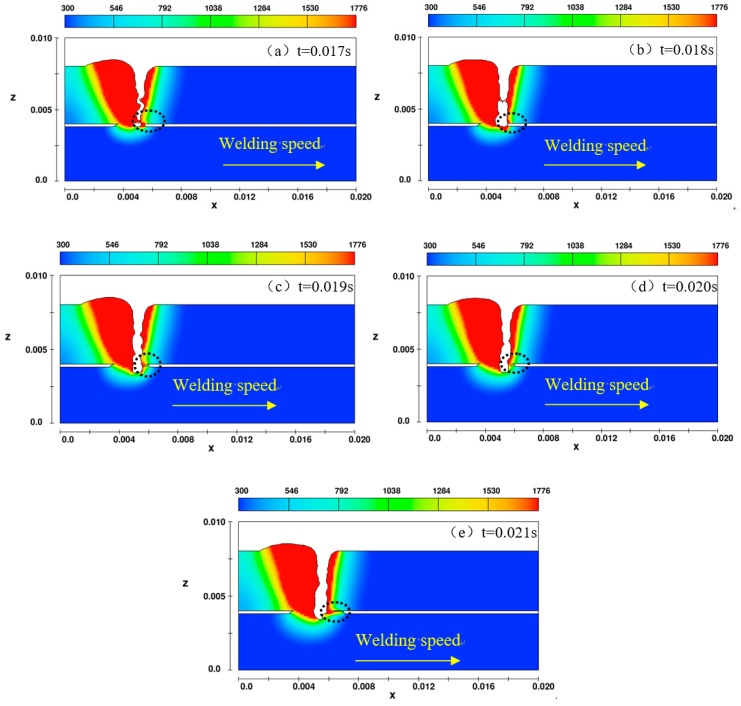
The pool connecting mechanism in the gap location along the x-z plane.

Figure 4 shows the profile along the x-z plane perpendicular to the welding direction. In the front of keyhole, the gap is always filled with the fluid and the keyhole in the side wall is closed. With the movement of keyhole, this welding process is the same as that without gap present. The air cannot be entrapped into the pool from the gap in front of keyhole. Hence, the presence of gap will not produce the effect on the pore formation because the pool in the gap is always continuous around the side wall.

According to the above discussion, the laser does not shine on the core directly after the welding is stable. The dropped fluid firstly removes the air around the gap to prevent it from entering the pool and forming pores. This connecting mechanism further implies that the small gap presence does not reduce the welding quality. However, when the gap size is ≥0.4 mm, worse effects of gap size on the welding quality will be produced, as shown in a previous paper [12].

### 3.2. The Velocity Field within the Molten Pool

**Figure 5:**
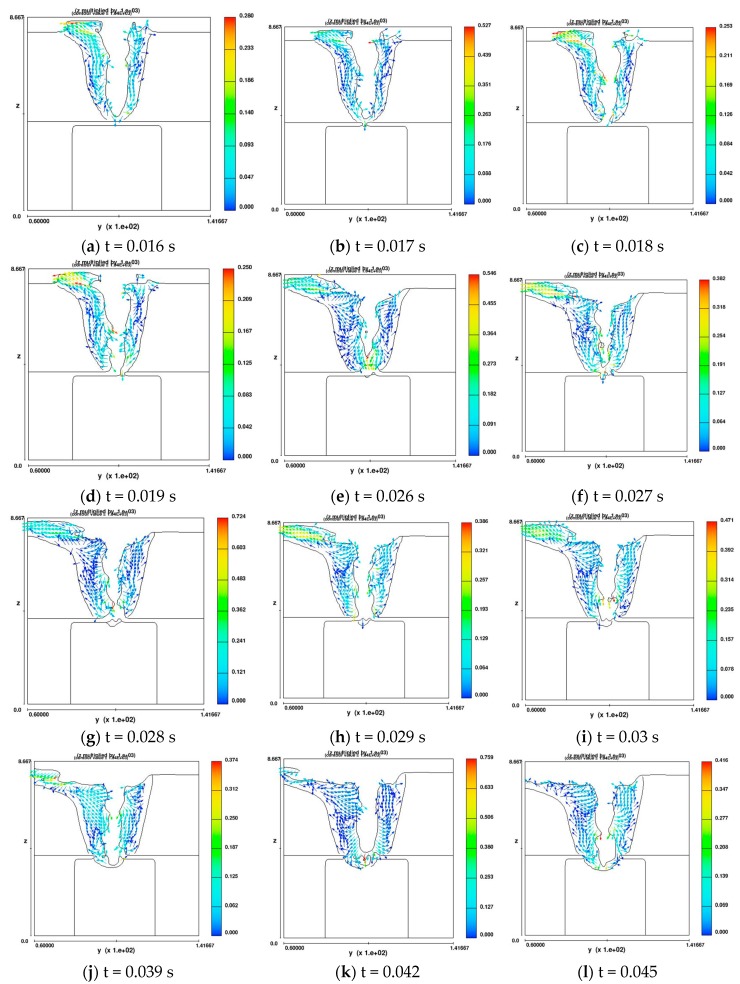
The velocity field corresponding to Figure 3.

To prove whether the gap presence will produce effects on the pores when the face sheet is penetrated, the corresponding velocity field is given in Figure 5. The fluid presents larger velocity in the surface of keyhole. At t = 0.018 s, the keyhole goes through the face sheet when the largest value of velocity in the pool gets smaller compared to without penetration. From t = 0.018 to t = 0.03 s, the velocity becomes smaller while the fluid quickly connects the face with core. The direction of velocity is down in the lower pool and up in the top pool during the entire penetration welding. This velocity field, which is similar to the full penetration, can drive all the air out of the pool. At t = 0.042 s, the face and core sheet connect and the maximum velocity locates in the original gap. Then, the maximum velocity gets smaller as the welding depth increases after connection.

Hence, the velocity field of fluid during penetrating the gap is similar to the full penetration, which keeps the keyhole fully open and produces few pores. After the pool connects the face with core sheet, this situation is the same as with non-gap welding.

### 3.3. Experimental Verification

**Figure 6:**
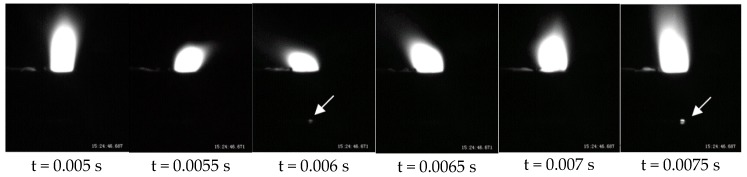
The plasma pattern under the gap presence of 0.2 mm.

Two experiments were conducted to verify the simulation. According the previous simulation, the pool was discontinuous during penetrating the gap. Thus, in the experimental welding process, some plasma was observed through the gap according to the pool repeatedly being continuous and discontinuous. When the pool was discontinuous, the plasma escaped from pool, which was captured by the camera. Figure 6 shows the plasma images with the gap presence of 0.2 mm. The gap is marked by the arrow. The plasma shown in the gap appeared discontinuously and could not always be seen. When the plasma was observed, it indicated that the pool is cracked (Figure 3). At this time, there is no fluid in the gap. Then, the plasma is not seen, indicating that the fluid surrounds the keyhole. Moreover, the frequency of connecting and disconnecting is 0.002 s, which is the same as the simulation. This phenomenon agrees well with simulated results and verifies the discontinuous connection of pool in the gap.

**Figure 7:**
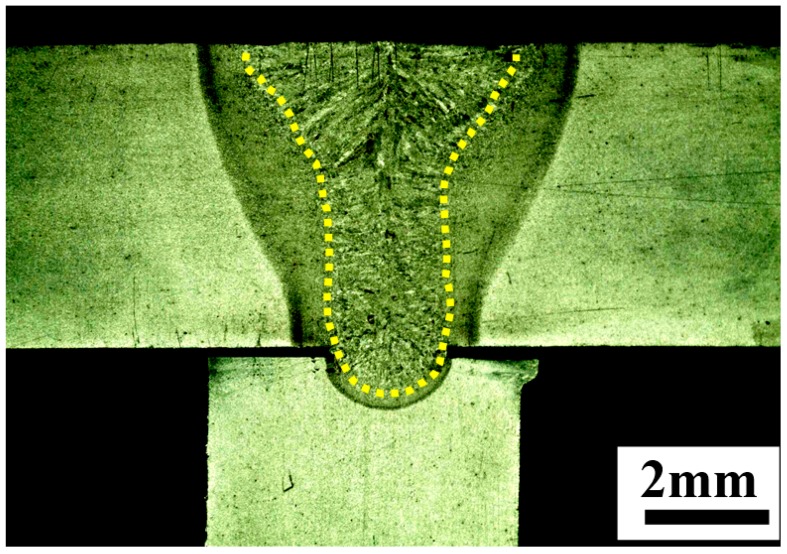
Cross sectional profile of welding bead without pores.

The other experimental verification was observing the cross-sectional welding performance under the simulated parameters. After finishing the welding with the same parameters, the cross-sectional profiles of welding bead were cut and observed using a Zeiss optical microscope (Carl Zeiss, Jena, Germany). Many cross-sections were polished and etched with nitric-alcohol solution, but few pores were found in the gap location, as shown in Figure 7. This also indicates the simulated thermal field is reasonable.

## 4. Conclusions

A three-dimensional physical model of laser welding T-type joint with the gap presence of 0.2 mm was built based on low carbon steel material. The connecting mechanism of pool between the face and the core sheet was obtained through simulating the thermal and flowing field in the gap location. The main conclusion can be drawn as follows.

(1) Minor gap within 0.2 mm will not reduce the welding quality. Under the effects of gravity and surface tension, the fluid from the face sheet will drop down to the core, which removes the air from the gap and the laser goes through the dropped fluid of the gap and then shines on the core which prevents the air from being entrapped into the pool.

(2) When the laser goes though gap, the wall of keyhole is opened and closed continuously. The vibrating time of keyhole is approximately 0.029 s (from t = 0.016 s to t = 0.045 s). After finishing the vibration, the welding is stable, which is the same as common unfull penetration.

(3) The simulated results are well verified through observing plasma phenomena and comparing the welding geometry and integrity. The frequency of discontinuous connection in the gap agrees well with the simulation. The weld performance with few pores proves that air cannot be entrapped into the pool to form large pores.

## Figures and Tables

**Table 1:** The physical parameters used in the simulation.

Solid Density/kg·m^−3^	Solid Specific Heat/J·kg^−1^·K^−1^		Solid Conductivity/W·m^−1^·k^−1^	Viscosity/Pa·s	Melting Point/K	Liquid Specific Heat/J·kg^−1^·K^−1^	Liquid Conductivity/W·m^−1^·k^−1^	Liquid Density kg·m^−3^		Surface Tension/N·m^−1^	Melting Latent Heat/J·kg^−1^
6990	710	27.2		0.0055	1776	680	26.7		7020	1.8	277,000
